# Antioxidant Activities of Stilbenoids from *Rheum emodi* Wall

**DOI:** 10.1155/2012/603678

**Published:** 2012-10-24

**Authors:** Yuan-yuan Chai, Fang Wang, Yan-li Li, Ke Liu, Hui Xu

**Affiliations:** School of Pharmacy, Yantai University, Yantai 264005, China

## Abstract

*Rheum emodi* Wall has been reported to possess protective effect in many inflammatory diseases and oxidative stress-related injuries. This study aims to investigate antioxidant power of stilbenoids from *R. emodi* and then explore the material basis for its antioxidant potential. The most abundant stilbenoid piceatannol-4′-O-**β**-D-glucopyranoside (PICG) and its aglycon piceatannol (PICE) were isolated from *R. emodi* rhizome. Using well-accepted antioxidant chemicals as reference, antioxidant activity of these stilbenoids was examined by measuring DPPH and superoxide anion radical scavenging, ferric reducing power, and inhibition of lipid peroxidation *in vitro*. Both PICG and PICE displayed promising antioxidant activity in all the four assays. Comparisons among the tested compounds indicated that PICE has the most potent antioxidant activity and the presence of 3′-hydroxyl group may enhance antioxidant activity of stilbenoids. The antioxidative effect of PICE at the cellular level was further demonstrated on the model of hydrogen-peroxide-induced H9c2 rat cardiomyoblasts injury. Taking into account the rapid *in vivo* metabolic transformation of PICG into PICE it can be inferred that the most abundant stilbenoid PICG may be an important constituent responsible for the antioxidant potential of *R. emodi* and promising to be developed as an antioxidant agent for supplementary or therapeutic use.

## 1. Introduction

**Figure 1:**
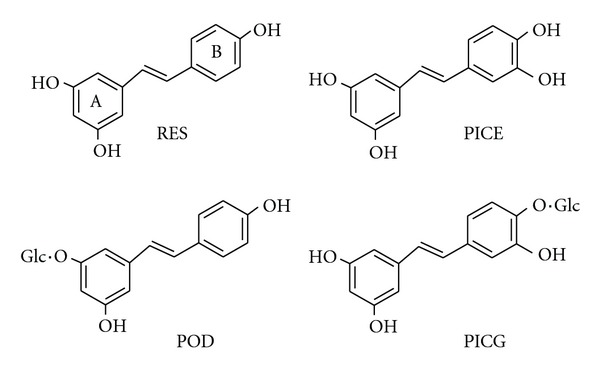
Chemical structures of the four stilbenoid compounds investigated. PICE and PICG were isolated from *R. emodi* rhizomes, whereas RES and POD were from commercial source and used as references for comparison.

Reactive oxygen species (ROS) are chemically reactive molecules containing oxygen. Examples include oxygen ions and peroxides. In biological system ROS form as a natural byproduct of the normal metabolism of oxygen and have important roles in cell signaling and homeostasis [1]. ROS are also generated by exogenous sources (such as ionizing radiation) and times of environmental stress (e.g., UV or heat exposure). This may result in dramatically increased ROS levels and then significant damage to cell structures [2]. Cumulatively, this is known as oxidative stress, which plays a key role in the pathophysiology of many age-related degenerative diseases such as age pigments, cataractogenesis, heart attack, stroke, liver injury, and cancers [3, 4]. Several endogenous antioxidant systems are developed in human body to balance the production of ROS. When endogenous antioxidants are insufficient, exogenous supplements would be necessary for preventing oxidative damages [5, 6]. During the last few years, vegetal stilbenoids have received considerable attention as source of antioxidants for various health benefits and safety in biological systems. Resveratrol (3, 5, 4′-trihydroxystilbene, Figure 1), a naturally occurring phytoalexin present in numerous plant species, is a representative. Large numbers of studies have demonstrated that this dietary chemical exerts various beneficial effects in organism and may be a useful chemoprotective agent of various important pathologies [7–9]. Recently, the major form of resveratrol in plants, its 3-O-*β*-D-glucoside, namely, polydatin, was approved by State Food and Drug Administration of China for clinical trial to treat cardiovascular diseases [10].

**Rheum emodi Wall**

(Polygonaceae) is a food plant with medicinal value restricted to the temperate, subalpine, and alpine zones of the Himalayas in altitudes ranging from 2,800 to 3,800 m [11]. In China, *R. emodi* is mainly distributed to the west of the line from Daxinganling Mountains, Taihang Mountains, Qinling Mountains, Dabashan Mountains to Yunnan-Guizhou Plateau [12]. The roots and rhizomes of *R. emodi* have been in use in traditional Chinese and Tibetan medicine for the past 2,000 years to treat piles, haemorrhage, gastroenteritis, nephritis, and other inflammatory diseases [13]. The recent findings from animal test and clinical trials further indicated the hypoglycemic activity and neuroprotective effect of this plant [14, 15]. *R. emodi *is known to contain several secondary metabolites, of which anthraquinones (such as emodin and rhein) are considered as the active ingredients [16]. It has been recently reported that extracts of *R. emodi* rhizomes have antioxidant and cytotoxic activities, and phenolic compounds might be responsible for these therapeutic properties [17]. By now several stilbenoids including piceatannol (3,5,3′,4′-tetrahydroxystilbene) and its glycosides have been found in this plant [18, 19]. The content of piceatannol-4′-O-*β*-D-glucopyranoside in *R. emodi* could even be up to 7.5% of the total dry weight for both wild and cultivated species [20]. However, the antioxidant effects of this abundant stilbenoid from *R. emodi* have not been reported yet.

The present study aims to investigate antioxidant potential of stilbenoids in *R. emodi*, and then explore the material basis responsible for the antioxidant activities of this herb. Piceatannol and its 4′-O-*β*-D-glucopyranoside were chromatographically isolated from 95% ethanol extract of *R. emodi* rhizome. Various methods were applied for antioxidant activity evaluation, including 1,1-Diphenyl-2-picrylhydrazyl (DPPH) and superoxide anion radical scavenging, ferric reducing power, inhibition of lipid peroxidation, and protection on cardiomyocyte injury induced by hydrogen peroxide (H_2_O_2_) *in vitro*. Along with ascorbic acid (an endogenous antioxidant) and edaravone (3-methyl-1-phenyl-pyrazolin-5-one, a radical scavenging agent used for patients with cerebral infraction), resveratrol and its glycoside polydatin were used as reference compounds for comparison and structure-activity analysis.

## 2. Materials and Methods

### 2.1. Chemicals and Other Reagents

DPPH, thiobarbituric acid (TBA), trichloroacetic acid (TCA), and dimethyl sulfoxide (DMSO) were purchased from Sigma Chemical Co. (St. Louis, MO, USA). Resveratrol (RES) and polydatin (POD) were supplied by Great Forest Biomedical Ltd. (Hangzhou, China). Edaravone (EDA) and ascorbic acid (Vc) were obtained from Aladdin Reagent Co. (Shanghai, China). Fetal bovine serum (FBS) and cell culture medium Dulbecco's Modified Eagle's Medium (DMEM) were purchased from Sijiqing Biological Engineering Materials Co. Ltd. (Hangzhou, China) and Invitrogen Corporation (USA), respectively. Penicillin, streptomycin, and antiactin were purchased from Beyotime Institute of Biotechnology (Jiangsu, China). Trypsin and 3-(4,5-dimethylthiazol-2-yl)-2,5-diphenyltetrazolium (MTT) were products of Amresco Corporation (USA). The remaining chemicals and solvents used were of standard analytical or HPLC grade.

### 2.2. Extraction, Isolation, and Identification

The air-dried *R. emodi* rhizomes were collected from the cultivation base of Tibetan medicinal materials in Lhasa (at altitude of 3600 m), Tibet, China in July, 2010. The rhizomes (1 kg) were powdered and then extracted with 95% ethanol under reflux (3 × 5 L, 2 h each time). The EtOH extract was concentrated under vacuum to yield crude extract, which was suspended in water and then successively extracted with petroleum ether (60–90°C, 2 × 2 L), EtOAc (2 × 2 L), and *n*-BuOH (2 × 2 L), respectively. The EtOAc solution was concentrated to give a residue for silica gel column chromatography using gradient mixtures of CHCl_3_-MeOH (1 : 0~0 : 1) as eluents. Then two compounds with the purity above 98% were yielded. By comparison of physical and spectral data with literature [21, 22], they were characterized as piceatannol (PICE) and piceatannol-4′-O-*β*-D-gluco-pyranoside (PICG), respectively. The chemical structures were shown in Figure 1.

### 2.3. HPLC Analysis

 The chemical profiles of various subsections from crude extract were analyzed by an Agilent 1100 HPLC system consisting of a G1314A variable wavelength detector and a G1312A binary pump, and equipped with an Agilent Zobax SB-C18 column (4.6 mm × 250 mm, 5 *μ*m). Gradient elution was performed at 30°C with solution A (methanol) and solution B (0.3% HCOOH in water) in the following gradient elution program: 0–20 min—33% of solution A; 20–25 min—46% of solution A; 25–30 min—70% of solution A. Detection was conducted at wavelength of 320 nm. Flow rate and injection volume were set at 1 mL/min and 20 *μ*L, respectively.

### 2.4. DPPH Radical Scavenging Assay

This assay protocol was modified from the microplate-based method as described by Fukumoto and Mazza [23]. Test samples were dissolved in DMSO and serially diluted into different concentrations. An aliquot of 10 *μ*L sample solution was mixed with 190 *μ*L fresh prepared DPPH ethanol solution (200 *μ*mol/L) on a 96-well microplate at room temperature. After incubation in dark for 30 min, DPPH level of each well was evaluated by detecting absorbance at 517 nm with Microplate Reader (BioTek Instruments Inc., USA). Percentage DPPH scavenging activity was calculated and expressed as %DRSA. 

### 2.5. Superoxide Anion Scavenging Assay

According to the instructions from manufacturer, a commercial kit (Jiancheng Biologic Engineering Co. Ltd., Nanjing, China) was used for superoxide anion (O_2_^−•^) scavenging activity assay. In order to mimic the xanthine and purine oxidase reaction system in organism, both electronic transmit substance and Gress reagents were applied to obtain superoxide anion radical. The solutions with various concentrations were accurately prepared for test. Absorbance at 550 nm was measured for sample tube and control tube, respectively. Then percentage superoxide anion scavenging activity was calculated and expressed as %SASA.

### 2.6. Lipid Peroxidation Assay

 This assay is based on the reaction of MDA with TBA forming an MDA-TBA_2_ adduct and performed as described by Zhou and Li [24] with slight modification. The peroxidation reaction system contained 200 *μ*L 1.5% lecithin in phosphate buffer (0.1 mol/L, pH 7.4), 100 *μ*L sample solution, and 200 *μ*L FeSO_4_·7H_2_O solution (25 mmol/L). Phosphate buffer was added to make a final volume of 2 mL. The reaction mixture was incubated at 37°C for 1 h. Then 1.75 mL of TCA solution (20%) was added to stop the oxidation reaction followed by an addition of 0.25 mL TBA (3.2% in 0.1 mol/L NaOH). The reaction solution was delivered to a boiling water bath for 20 min, cooled and centrifuged at 3500 g for 10 min. Absorbance of the supernatant was recorded at 532 nm. Percentage inhibition of lipid peroxidation was calculated and expressed as %LPI.

### 2.7. Ferric Reducing Power Assay

This assay was conducted according to the protocol of Oyaizu [25] with some modifications. An aliquot of 1.0 mL test sample was mixed with 2.5 mL phosphate buffer (0.2 mol/L, pH 6.6) and 2.5 mL 1% aqueous solution of potassium ferricyanide. The mixture was incubated in a water bath at 50°C for 20 min, then 2.5 mL of TCA solution (10%, W/V) was added followed by centrifugation at 600 g for 10 min at room temperature. An aliquot of 0.25 mL supernatant was collected and then combined with 3.75 mL distilled water and 1.0 mL fresh-prepared ferric chloride solution (0.1%, W/V). Absorbance of the reaction mixture was measured immediately at 700 nm. According to standard calibration curve, the amount of Fe^2+^ was calculated. Then concentration-effect relationship was plotted, and linear regression was conducted. With Vc as reference, the slope ratio was further calculated as ascorbic acid equivalence (AAE) to quantitatively compare ferric reducing power. The reducing power of 1 AAE means that the reducing power of 1 *μ*mol/L sample is equivalent to 1 *μ*mol/L ascorbic acid.

### 2.8. Cell Culture, Treatments, and MTT Assay for Cell Cytotoxicity

This assay was performed according to the protocol as previously described [26]. Normal rat cardiomyocyte H9c2 cells (Cell Bank of Chinese Academy of Science, Shanghai, China) were maintained in DMEM medium supplemented with 20% FBS, 100 U/mL penicillin, and 100 *μ*g/mL streptomycin at 37°C in a humidified atmosphere of 5% CO_2_ and 95% air. After being cultured for 24 h, cells were pretreated with or without various concentrations of test samples for another 24 h, followed by incubation with H_2_O_2_ (120 *μ*mol/L) for 1 h. Cells then were transferred to 96-well plates at a density of 1 × 10^5^ cells/well. The number of surviving cells was counted using the MTT assay, through MTT labeling at a final concentration of 0.5 mg/mL for another 4 h incubation at 37°C. The reduced MTT-formazan was solubilized with 100 *μ*L of DMSO, and the absorbance of MTT-formazan solution at 570 nm was measured with Microplate Reader (BioTek Instruments Inc., USA) using 630 nm as reference wavelength. The cell viability was calculated according to the ratio of absorbance value of sample-treated cells to that of nontreated cells.

### 2.9. Statistical Analysis

All data *in vitro* represent the mean of samples from three separate experiments. Results were expressed as mean ± standard deviation. The significance of difference was analyzed by one-way ANOVA followed by Tukey's test. A value of *P* < 0.05 was considered statistically significant.

## 3. Results

### 3.1. Estimation of PICE and PICG Contents in *R. emodi* Rhizomes

With the isolated compounds as control, contents of PICE and PICG in various subsections from extract of *R. emodi* rhizomes were determined. As shown in Table 1, EtOAc-soluble fraction was the most abundant fraction for both PICE and PICG, while petroleum-soluble fraction contained the least amount of stilbenoids. Moreover, the content of PICG was 10 times more than that of PICE in EtOAc-soluble fraction or *n*-BuOH-soluble fraction. In EtOAc-soluble fraction the contents of PICG and PICE were 12.92% and 0.86%, respectively.

### 3.2. Scavenging Activities against DPPH and O_2_^−•^ Radicals

**Figure 2:**
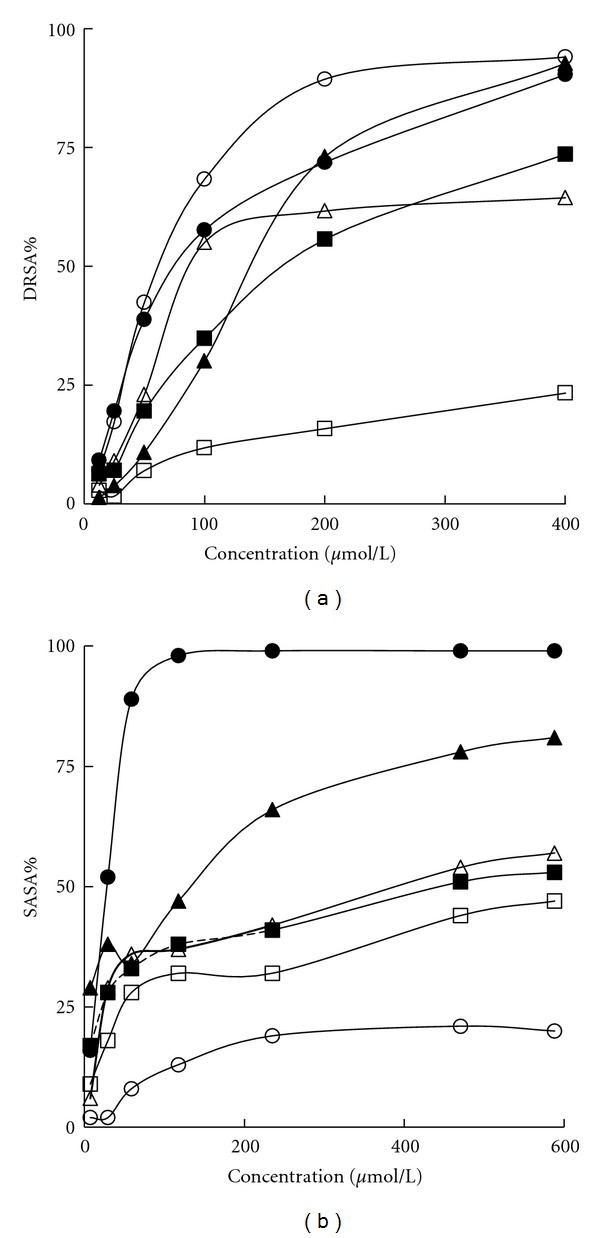
Scavenging effect on DPPH radical (a) and superoxide anion (b). ▲, ∆, ■, □, ●, and ○ indicated PICE, PICG, RES, POD, Vc, and EDA, respectively. Results are presented as the mean scavenging percentage (DRSA% for DPPH radical and SASA% for superoxide anion) from triplicate tests.

For both DPPH and O_2_^−•^ radicals, all the four stilbenoid compounds displayed a concentration-dependent scavenging. Results were plotted as %DRSA and %SASA and illustrated in Figures 2(a) and 2(b), respectively. The scavenging capacity was further expressed as IC_50_ value (the concentration causing 50% inhibition) and shown in Table 2. As to DPPH radical scavenging, PICE was found to be more efficient than the other three stilbenoids, of which POD was the weakest and merely showed 29% scavenging at 0.8 mmol/L. However, all the four stilbenoids were weaker than Vc and EDA. The two reference compounds showed similar scavenging ability against DPPH radical with the IC_50_ values of 77.52 *μ*mol/L and 62.63 *μ*mol/L for Vc and EDA, respectively. As for superoxide anion radical, Vc displayed the strongest scavenging with a IC_50_ value of 18.97 *μ*mol/L, while EDA was the weakest with 15% scavenging at 0.6 mmol/L. All the stilbenoids displayed a scavenging tendency similar to that against DPPH radical. At a concentration of 0.6 mmol/L, PICE, PICG, RES, and POD showed 78%, 47%, 43%, and 39% scavenging, respectively. 

### 3.3. Lipid Peroxidation Assay

**Figure 3:**
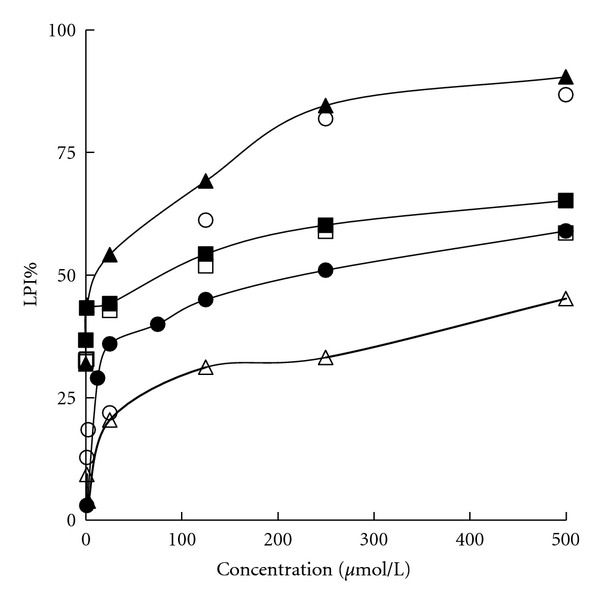
Inhibitory effect on lipid peroxidation. ▲, ∆, ■, □, ●, and ○ indicated PICE, PICG, RES, POD, Vc, and EDA, respectively. Results are presented as the mean scavenging percentage (DRSA% for DPPH radical and SASA% for superoxide anion) from triplicate tests and expressed as LPI%.

The results of antilipid peroxidative effect plotted as %LPI were illustrated in Figure 3. The inhibitory capacity expressed as IC_50_ value was further shown in Table 2. All the tested compounds but PICG were capable of preventing formation of MDA in a concentration-dependent manner. PICE was observed to be the most potent inhibitor of lipid peroxidation with an IC_50_ value slightly less than that of EDA. For stilbenoids, both PICE and RES were significantly more potent than their glycoside, PICG and POD, respectively.

### 3.4. Ferric Reducing Antioxidant Property

**Figure 4:**
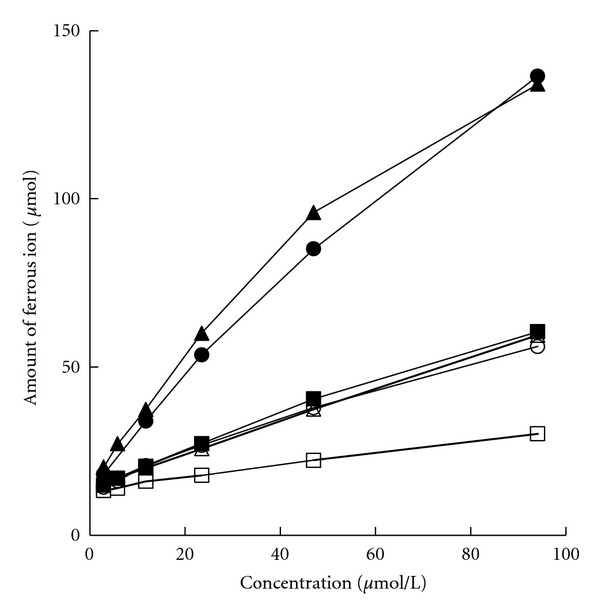
Ferric reducing power. ▲, ∆, ■, □, ●, and ○ indicated PICE, PICG, RES, POD, Vc, and EDA, respectively. Results are presented as the mean amount of ferrous ion produced in triplicate tests.

Ferric reduction property of all the stilbenoids and reference compounds were displayed as the amount of Fe^2+^ in Figure 4. AAE value for further quantitative comparison was shown in Table 3. All the test compounds exerted ferric reduction property in a concentration-dependent manner. Moreover, PICE showed the highest reducing power and was even more efficient than Vc. PICG had an AAE value of 0.494, which was much lower than that of PICE (1.195) but close to those of RES (0.532) and EDA (0.435). POD displayed the weakest reducing power with an AAE value of 0.195.

### 3.5. Effect of PICE on H_2_O_2_-Induced Deficiency of Cell Viability

**Figure 5:**
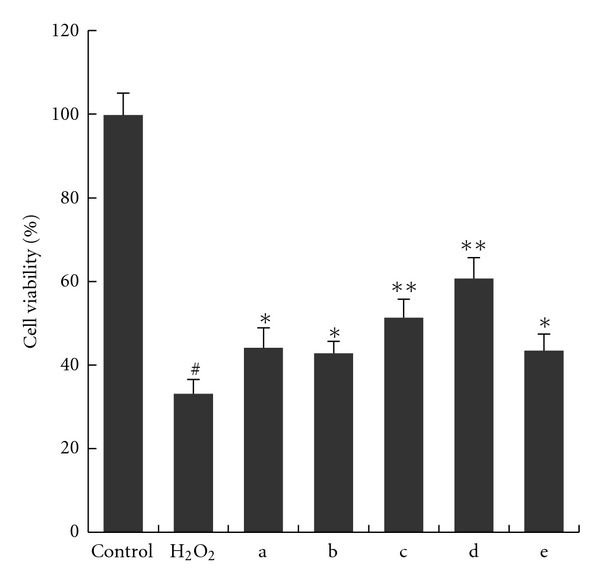
Protective effect of PICE against H_2_O_2_-induced oxidative damage in rat cardiomyoblast H9c2 cells. Cells were pretreated with or without test compound for 24 h prior to incubation with H_2_O_2_ for 1 h. Data are represented as mean ± SD of triplicate tests. Group a was pretreated with 20 *μ*M EDA, and groups b to e were pretreated with PICE under concentration of 0.04, 0.2, 1, and 5 *μ*M, respectively. ^#^ *P* < 0.01 versus control group; *P < 0.05, **P < 0.01 versus H_2_O_2_ group.

Using the model of H9c2 cells induced by H_2_O_2_, the protective activity against oxidative damage was further evaluated for PICE, the most potent stilbenoid in the above four *in vitro *antioxidant assays. Moreover, edaravone (EDA) was used as a positive control since its protective effect on H9c2 cells against chemical hypoxia induced injury has been reported [27]. As shown in Figure 5, H9c2 cells viability fell to 32.0 ± 4.3% with exposure to 120 *μ*M H_2_O_2_ for 1 h. When preconditioned with 20 *μ*M EDA, the inhibition of cell viability by H_2_O_2_ was effectively blocked. Pretreatment with PICE could significantly attenuate the cytotoxicity of H_2_O_2_ and increase cell viability in a dose-dependent manner at concentrations ranging from 0.04 *μ*M to 1 *μ*M, which suggested that PICE could decrease H_2_O_2_-induced oxidative damage to protect cardiomyocytes.

## 4. Discussion

A growing amount of evidence indicates that ROS are associated with aging and various degenerative diseases such as cancers, cardiocerebrovascular diseases, Alzheimer's and Parkinson's diseases [28]. The antioxidants can protect from the potentially damaging oxidative stress, which is a result of an imbalance between the over formation of ROS and body antioxidant defense [29]. During the last few years, interest has considerably increased in finding naturally occurring antioxidants for the potential in health promotion and disease prevention, and the high safety and consumer acceptability [30]. Plant secondary metabolites have been extensively studied in the recent search of novel sources of antioxidants, among which floral stilbenoid is one of the most promising class. 

In the present study, PICE and its glucoside PICG from *R. emodi* were found to possess promising antioxidant activity in various *in vitro* models used. When compared with the reference stilbenoids, PICE was observed to be more active than RES, and PICG more active than POD. On the other hand, both PICE and RES were more potent than their respective glycosides, PICG and POD. These results indicated that antioxidant activity of stilbenoid compounds might increase with the number of hydroxyl group. Moreover, the presence of 3′-hydroxyl group would significantly enhance antioxidant activity. It was consistent with the concept that antioxidant properties of stilbenoids are associated with their ability to form stable radicals, and 3′-hydroxyl group could stabilize the semiquinone radical-anion intermediate by resonance through the *trans* double bond [31]. This might be further demonstrated by the fact that aromatic compounds with catechol structure appear to be important scavenger [32]. 

It is well known that reducing power is related to the ability to transfer electrons and may, therefore, serve as an important index of potential antioxidant activity. Vc is just a vital endogenous antioxidant for prominent reducing property. With an AAE value exceeding 1.0, PICE was observed to be the most potent reducer among the four stilbenoids investigated, and even more potent than Vc. This stilbenoid was also revealed to strongly inhibit lipid peroxidation with an IC_50_ value close to that of edaravone (EDA), which is the only free radical scavenger within the past decade clinically used as neuroprotective agent with protecting against lipid peroxidation as the main mechanism [33, 34]. Lipid peroxide is a free-radical chain reaction accelerated by ROS. The ability to prevent lipid peroxidation might directly relate to the quenching of hydroxyl radicals and then the generation and development of many diseases [35, 36]. These findings suggest that PICE might prevent reactive radical species from damaging biomolecules such as lipoproteins, polyunsaturated fatty acids, DNA, amino acids, proteins, and sugars in biological systems. 

Pharmacokinetic studies on these stilbenoids from *R. emodi* have recently been carried out to understand their *in vivo* process. It is indicated that PICG administrated to rats by oral or vein could be quickly metabolized (within 5 min after administration) and PICE is a principal metabolite [37]. Cleaving the glycosidic bond through enzymatic or bacterial hydrolysis is a prevalent metabolic pathway for glycosides. Polydatin is an example and reported to be metabolized into resveratrol after intravenous administration in dog and rat [38]. Although PICG showed inferior antioxidant capability to its aglycon PICE in all these *in vitro* assays, the rapid metabolic transformation combining with the potency of its metabolite thus may make the naturally abundant stilbenoid PICG a promising antioxidant agent *in vivo*. 

In order to demonstrate antioxidant potency of PICE at the cellular level, we further evaluate protective effect of PICE on H_2_O_2_-induced oxidative injury in H9c2 cells, a cardiac myoblast cell line derived from rat heart tissue. Super-physiologic increases in ROS can cause oxidative injury to tissue cells. Among various activated oxygen species, H_2_O_2_ plays a significant role in oxidative stress injury and is a well-accepted model to induce ROS and oxidative damage [39]. Data from the present study indicated that PICE could effectively protect H9c2 cells from oxidative damage in a dose-dependent manner within certain concentration (ranging from 0.04 *μ*M to 1 *μ*M). Importantly, these findings provided much more convincing evidence to develop this natural product for therapeutic use. For subsequent research, we need to carry out more relative experiments on this compound to determine its *in vivo *antioxidative characteristics and the precise molecular mechanism involved. 

In conclusion, all the results from the present study support that* R. emodi *is a medicinal herb with potent antioxidative activity and its effects may contribute to the stilbenoids in this plant. As the most abundant stilbenoid compound in *R. emodi *rhizomes, PICG is worthy to be developed as a beneficial antioxidant since it could be rapidly *in vivo* metabolized into PICE with outstanding antioxidant potency, especially in reducing power, preventing lipid peroxidation, and decreasing oxidative stress-induced cell damage. 

## Figures and Tables

**Table 1:** Weight percentage (%) of PICE and PICG in different subsections of *R. emodi* rhizome extract.

	Petroleum	EtOAc	*n*-BuOH
PICE	0.023	0.86	0.12
PICG	0.045	9.92	6.48

**Table 2:** Antioxidant activities of different compounds expressed as IC_50_ values (*μ*mol/L) from *in vitro* assays of DPPH (I) and superoxide anion (II) scavenging and lipid peroxidation inhibition (III).

	PICE	PICG	RES	POD	Vc	EDA
I	138.9	149.8	167.2	—	77.5	62.6
II	124.6	305.2	438.3	—	18.97	—
III	31.28	—	37.70	72.89	166.4	29.65

— indicated that the activity was rather weak and therefore IC_50_ value could not be calculated.

**Table 3:** Linear regression analysis of dose-response data from ferric to ferrous reduction assay for different compounds.

	PICE	PICG	RES	POD	EDA	Vc
Slop	1.2535	0.4839	0.5018	0.1825	0.4548	1.2267
Intercept	23.779	14.191	14.599	13.273	14.615	20.764
*r *	0.9966	0.9998	0.9968	0.9973	0.9952	0.9966
AAE	1.022	0.394	0.409	0.149	0.371	1.000

AAE, namely, ascorbic acid equivalence, is the slope ratio calculated with Vc as reference.
